# Expected long-term defect rate of analytical performance in the medical laboratory: Assured Sigma *versus* observed Sigma

**DOI:** 10.11613/BM.2018.020101

**Published:** 2018-06-15

**Authors:** Hassan Bayat

**Affiliations:** Immunogenetics Research Center, Mazandaran University of Medical Sciences, Sari, Iran

**Keywords:** medical laboratory, Sigma metrics, Six Sigma, allowable total error

## Abstract

Reliability of laboratory results is determined by the ratio of incorrect results expected in long-term. Sigma is a measure of defect ratio, therefore long-term Sigma is a measure of the reliability of laboratory results. Commonly, long-term Sigma is estimated based on the short-term Sigma. The Six Sigma methodology assumes that in long-term performances will shift up to 1.5 Sigma, and therefore the long-term Sigma is considered 1.5 Sigma less than short-term Sigma. Analytical performance in the medical laboratory is prone to shifts larger than 1.5 Sigma. Thus, the 1.5 Sigma shift assumed in the Six Sigma is not a correct estimate in the medical laboratory. On the other hand, in the medical laboratory statistical quality control procedure (SQC) is applied to detect and correct shifts. Since SQC can be planned to trap shifts of different sizes, the threshold set for SQC determines the defect rate expected for long-term.

## Introduction

“Medicine is a science of uncertainty and an art of probability” - William Osler (*1*). It is the same with medical laboratory results, and the results cannot be of complete reliability; but must be of enough reliability to let clinical decisions be made with low uncertainty and high probability. In the medical laboratory, the quality requirement is usually defined in terms of total allowable error (TEa). Results that contain analytical errors that exceed TEa are considered incorrect results or defects. The reliability of the results is determined by the ratio of incorrect results produced by the assay method; *i.e.* the defect rate. The higher is the defect rate, the lower is the reliability and *vice versa*. For example, if a performance produces 5% incorrect results, then there is a 95% probability that any certain result is correct.

The defect rate can be determined either (a) directly by counting the defects, or (b) indirectly by calculations based on the Gaussian (normal) distribution as the area under the curve beyond allowable limits. In 1986, the Motorola Company introduced the Six Sigma concept as a quality management technique (*2*). The Sigma score or Sigma metrics is the core measure in the Six Sigma methodology and provides an indirect estimate of defect rate (*2*). The term Sigma implies to the imprecision of the performance and presents the number of standard deviations between mean and tolerance limit (TL). The original industrial equation for calculating Sigma is: Sigma = (TL – |Shift|) / SD, where TL represents the maximum allowable deviation from the target value; Shift is the difference between mean and target value; and SD, standard deviation, represents the imprecision (*2*). Therefore, Sigma is the number of the SDs that fit in the distance between mean and the nearest TL to the mean.

Given the above equation, Sigma is in fact the normalized z-value at the tolerance limit. Using the characteristics of the Gaussian curve, the z-value is converted to probability (P) corresponding to the ratio of defects. The higher the Sigma (z-value) is, the less area is out of allowable limits and thereby the lower is the defect rate. Using Sigma to estimate defect rate has gained a widespread acceptance and different tools and tables to convert Sigma to defect rate are presented in various sources (*3*).

Defect rate is usually presented as defects *per* million (DPM) (or defects *per* million opportunities, DPMO). Usually, defect rate is determined via short-term evaluation, and then the expected long-term defect rate is estimated. In practice, the various influence factors affecting a performance cannot be completely evaluated in short-term evaluation, and therefore it is expected that the defect rate in the long-term is higher than the defect rate observed in the short-term; therefore the quality that can be assured for the long-term is estimated less than the quality observed in the short-term. Based on the Six Sigma methodology, short-term Sigma is determined from short-term evaluation; a figure of 1.5 is subtracted from the calculated Sigma to estimate the long-term Sigma; and then the long-term Sigma is used to calculate the long-term defect rate (*2*). This approach to calculating the long-term defect rate is usually applied in the medical laboratory for the estimation of the defect rate of analytical phase. This paper is going to argue this application and show that the long-term defect rate in the analytical phase in the medical laboratory is dependent on the capability of the quality control procedures; and therefore, the reliability of the results in long-term is determined by the threshold set for the control procedures.

## Sigma of analytical phase

The Six Sigma concept has been applied in different industries including health care. Since in the medical laboratory the defect rate is reflecting the reliability of the results, Sigma value can provide a good measure of the reliability of laboratory results. In recent years, the Six Sigma concept has gained increasing application in the medical laboratory; for example, as a valuable tool in evaluating the quality of analytical performance, planning appropriate quality control procedures, and improving the quality of testing process (*4*).

**Figure 1:**
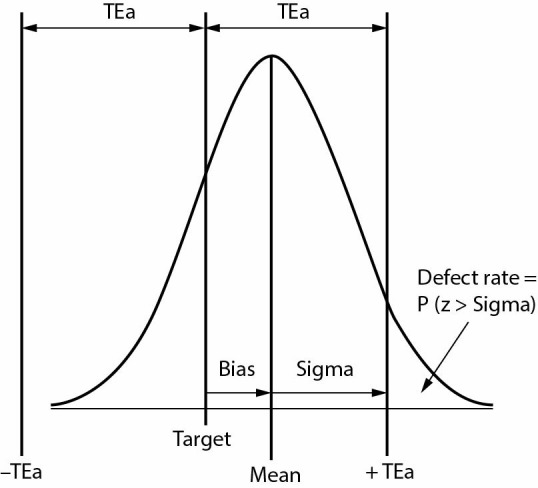
A schematic representation of defect rate resulting from bias and imprecision. Sigma value is the distance between mean and the nearest allowable total error (TEa) limit presented as multiples of standard deviation (SD). Defect rate is the area out of TEa limit. Here, the method is significantly biased, and therefore the one-sided probability is considered (the tail out of – TEa limit is very thin and is neglected).

In the medical laboratory, defect is defined as an erroneous result, which differs from its corresponding true value more than TEa. Given the terms ‘tolerance limit’ and ‘shift’ used in the industrial Sigma equation are respectively analogous to the TEa and bias of the analytical performance in the medical laboratory, J. O. Westgard adapted the industrial Sigma equation and introduced the following Sigma equation for laboratory application: Sigma = (TEa – |B|) / SD, where B is bias, and SD is the standard deviation (*4*). Using this equation, the ratio of incorrect results, or the analytical defect rate resulting from combined effects of bias and imprecision, can be estimated. The Sigma value as the z-value of the distribution at TEa is used for calculating the analytical defect rate: Defect rate = P (z > Sigma), where P is the probability that results falls out of TEa limits; *i.e.* the ratio of incorrect results or defect rate. With unbiased performances, the two-sided probability is considered, and the areas out of both TEa limits are summed to determine defect rate. With biases of ≥ 1 SD, the one-sided probability is considered, and the area out of the nearest TEa limit to mean represents the defect rate (the area out of the farthest TEa limit is neglected). Figure 1 shows the defect rate for a biased method. For the sake of ease, hereinafter in this paper defect rates are calculated using one-sided probability. For example, with a TEa of 7, a bias of 1 and SD of 2, Sigma = (7, 1) / 2 = 3. The Defect rate = P (z > 3) = 0.001349 ≈ 1350 DPM.

## Short-term defect rate *versus* long-term defect rate

Ideally, to determine the defect rate of a system, it must be evaluated for a substantially long period; preferably for the whole lifetime of the system. Given financial and time constraints, in practice the system is evaluated during a short period, defect rate is calculated from the short-term data, and then it is decided what defect rate is expected for long-term performance (*2*). If there were a 100% certainty that the performance observed in short-term would be completely stable in future, then it could be expected that the long-term defect rate is exactly the same as observed in the short-term (*2*). In practice, the systems tend to shift away from the stable performance, and, on the other hand, it is impossible to detect shifts instantly; therefore, it is expected that in long-term the defect rate is greater than short-term (*2*, *5*).

By convention established at Motorola, where the Six Sigma program originated, the Sigma level is adjusted by 1.5 sigma to recognize the tendency of processes to shift over the long term (*3*). It is assumed that in long-term there will be unpreventable shifts of different sizes - from zero to 1.5 SD - that will return to stable state spontaneously. The 1.5 SD shift is the worst case and the maximum decrease in quality that is expected for long-term. Given the worst case of 1.5 SD shift, Sigma expected for long-term is calculated as *Sigma_(long-term)_ = Sigma_(short-term)_ - 1.5*. For example, with a short-term bias of 1 and SD of 2 obtained from a method validation experiment, and TEa of 9, the calculation is as follows:

Sigma_(short-term)_ = (9 – 1) / 2 = 4

Defect rate_(short-term)_ = P (z > 4) = 0.0000317 ≈ 32 DPM

and

Sigma_(long-term)_ = Sigma_(short-term)_ – 1.5 = 2.5

Defect rate_(long-term)_ = P (z > 2.5) = 0.006209 ≈ 6200 DPM.

Meaning, based on the Six Sigma convention, with an observed quality of 4 Sigma (32 DPM) in short-term, the expected quality for long-term is 2.5 Sigma (6200 DPM). In this example, the 32 DPM is the error ratio that has occurred in *past*, and 6200 DPM is the maximum error ratio that is expected for *future*. In other words “the 1.5 Sigma shift indicates that if you intend to have 6200 DPM over the long term, the process must be more capable than the 2.5 Sigma in order to accommodate instabilities or process shifts that occur over time” (*3*). Note that this does not mean that the long-term defect rate for this example will always be 6200 DPM; but in long-term there will be shifts from zero to 1.5 SD; consequently the long-term defect rate will be between 32 DPM (for the shift of zero) and 6200 DPM (for the shift of 1.5 SD). When it is to assure the end user, the worst case scenario, *i.e.* the quality of 6200 DPM, is considered.

Sigma tables are constructed considering the assumed 1.5 Sigma shit. Table 1 shows an example Sigma table in which the defect rate presented for *e.g.* a short-term Sigma of 4 is 6200 DPM that is calculated as P (z > 2.5) instead of P (z > 4) to consider 1.5 Sigma shift over long-term (*3*).

**Table 1:** Example of Sigma table

**DPM**	**Short term Sigma**	**Long term Sigma**
**3.4**	6	4.5
**32**	5.5	4
**230**	5	3.5
**1350**	4.5	3
**6200**	4	2.5
**22,800**	3.5	2
**66,800**	3	1.5
**159,000**	2.5	1
**308,000**	2	0.5
**500,000**	1.5	0
**690,000**	1	- 0.5
DPM – defects per million. The defect rates are calculated after subtracting 1.5 from short-term Sigma to present the defect rate that is expected for long-term performance (*2*).		

## Long-term defect rate of analytical performance in medical laboratory

Concerning medical laboratory, it is worthy to ask: is 1.5 SD the maximum shift expected for analytical performance? And, do the shifts return back to the stable state spontaneously?

The limitation with the assumed 1.5 SD shift is that it is only a rule of thumb and “may or may not be an accurate estimate of the actual long-term instability of your process” (*3*). In fact, “the 1.5 value is a source of criticism in the literature because of its arbitrary (empirical) nature. (…) It is just a way of stating that processes are not stable forever, and this behaviour should be modelled somehow. This choice has proven to be a useful way to think about process performance." (*2*). The real world performances are prone to shifts substantially larger than 1.5 SD that are not corrected spontaneously. This is the case with the analytical performance in the medical laboratory. Analytical performance in the medical laboratory can be affected by various error sources causing shifts significantly larger than 1.5 SD, and in addition, there is no guarantee that the shifts will be corrected spontaneously. Therefore, the 1.5 SD can “not be an accurate estimate of the actual long-term instability” of analytical performance in the medical laboratory (*3*). On the other hand, since the shifts are not reversed by themselves, control procedures are needed to detect and correct instabilities before affecting patient safety (*5*).

**Figure 2:**
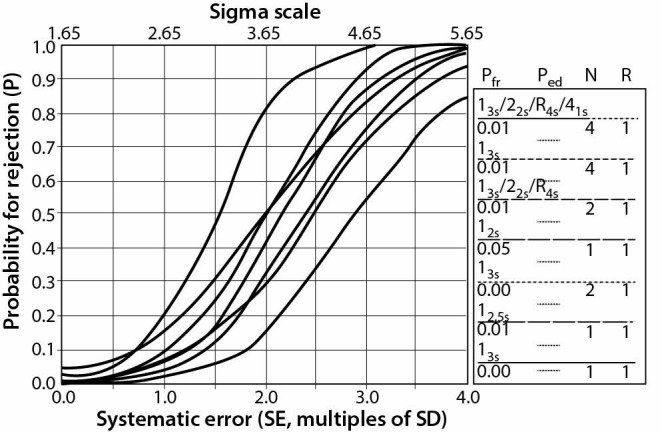
An example power graph for detecting the systematic error (SE). The characteristics of the statistical quality control (SQC) procedures in Table 2 are presented in this figure. The probability for rejection (P) is plotted on the y-axis *vs*. the size of systematic error on the lower x-axis (as multiples of SD) and *vs.* the Sigma on the upper x-axis. Power curves (top to bottom) correspond to SQC procedures in the key at the right (*4*). Pfr - the probability of false rejection. Ped - the probability of error detection. N - number of quality control material assayed per run. R - number of runs through which the SQC rule is interpreted. (The figure is produced by EZ Rules v.3 software. Westgard QC, Madison, USA).

A variety of statistical quality control (SQC) planning tools have been developed to detect shifts from the stable performance. The detection ability of SQC procedures is presented in power graphs in which probability of rejection is plotted *versus* the size of the error. Figure 2 is an example power graph for detecting the systematic error, SE. The probability of rejection or error detection (Ped) for SQC procedures is depended on the number of controls (N), the width of rejection borders, and the rules for interpreting quality control (QC) results. High-sensitive SQCs such as multi-rules with N of 4 to 6 can detect small shifts with a high Ped, while low-sensitive SQCs such as single rules with N of 1-2 can only detect large shifts with a high Ped (*5*).

Given different capabilities of SQCs for detecting shifts, applying different SQCs to the same performance leads to assuring different defect rates for long-term. Meaning, in obvious contrast to the Six Sigma methodology, the expected maximum long-term shift is not the arbitrarily assumed 1.5 Sigma, but it is depended on the capability of the applied SQC; the less sensitive is the SQC, the larger is the shift that goes on undetected. Therefore, referring to usual Sigma tables (*e.g.* Table 1) for determining long-term defect rate will be misleading.

In laboratory application of Sigma, it will be helpful that the terms "short-term Sigma" and "long-term Sigma" be respectively considered as "Observed Sigma" and "Assured Sigma":

Observed Sigma: Sigma calculated from short-term data, *e.g.* from method validation experiment;
Assured Sigma: The least expected Sigma for long-term, that is depended on the capability of the applied SQC to detect shifts.

As extreme examples, with a very loose SQC, even very large shifts would go on without being detected and therefore maximum defect rate expected for long-term would be very larger than short-term. On the other hand, with a very powerful SQC that can detect very small shifts instantly, maximum defect rate expected for long-term is approximately the same as short-term.

In practice, there is no practical SQC to catch small shifts instantly, and therefore, the quality assured for long-term will always be somewhat less than the quality observed in short-term. The maximum decrease in Sigma expected for long-term is determined by the size of the shift that is needed for SQC to reach a high Ped. Although it is ideal to apply SQCs with Ped = 100%, such SQCs will increase the probability of false rejection (Pfr). In practice, SQCs are planned so that a Ped ≥ 90% is achieved while keeping Pfr ≤ 5% (*5*). The shift size needed for SQC to reach a Ped = 90% is called QC budget (*5*). QC budget is the Sigma consumed by SQC; denoted as Sigma_(SQC)_. Thus, the least Sigma expected for long-term can be calculated as:

Sigma_(Assured)_ = Sigma_(Obsserved)_ – Sigma_(SQC)_.

In the medical laboratory, when establishing a method, imprecision and bias are first determined via short-term method validation experiments (usually in 20 days) and then short-term Sigma is calculated (*5*). Thereafter, periodically (*e.g.* every 3 months) imprecision is revised using internal QC data, and bias is revised using external QC data (*5*). For example, if a method validation experiment has determined a bias of 0.5 and SD of 1, given TEa = 6,

Sigma_(Observed)_ = (6 – 0.5) / 1 = 5.5

Defect rate_(Observed)_ = P (z > 5.5) ≈ 0.02 DPM.

Here, the 0.02 DPM is the defect rate that has happened during the evaluation period, but the maximum defect rate that can be assured (expected) for long-term depends on the detection ability of the applied SQC. The rejection probability of, *e.g.*, 1:3s/2:2s/R:4s/4:1s N4 (the topmost SQC procedure in Figure 2) reaches 90% at approximately SE = 2.3 SD. That is 2.3 SD of the Observed Sigma will be consumed before this SQC catches the shift; *i.e.* Sigma_(SQC)_ = 2.3. If we apply this SQC to a 5.5 Sigma method, the performance will be rejected by 90% probability when the quality has decreased from 5.5 Sigma to 3.2 Sigma (5.5 – 2.3). Thus, by applying this SQC to a 5.5 Sigma performance, we can give assurance that the worst quality in long-term is 3.2 Sigma corresponding to 690 DPM; calculated as:

Sigma_(Assured)_ = Sigma_(Obsserved)_ – Sigma_(SQC)_ = = 5.5 – 2.3 = 3.2

Defect rate_(Assured)_ = P (z > 3.2) ≈ 690 DPM

Note that this does not mean that in long-term the defect rate will always be 690 DPM, but this is the worst defect rate that is expected to happen in long-term without being detected by the SQC.

If less sensitive SQCs are applied to this same 5.5 Sigma method, shifts larger than 2.3 SD are needed to reach 90% rejection probability. For example, 1:3s/2:2s/R:4s N2 will reject the performance with 90% probability at SE = 3.1 SD (Figure 2), *i.e.* Sigma_(SQC)_ = 3.1. With this SQC, the Assured Sigma is 2.4 (5.5 – 3.1) corresponding to 8200 DPM. To compare, based on the Six Sigma assumption (Table 1), the worst expected scenario for a 5.5 Sigma performance in long-term is 4 Sigma corresponding to 32 DPM which is very less than the defect rates calculated for the mentioned SQCs (690 DPM and 8200 DPM).

## The long-term quality targeted/assured by SQC

Commonly it is expected that the laboratory results are at least 95% reliable (*5*). To provide such a reliability, the defect rate should be ≤ 5%. Therefore, a defect rate of 5% (50,000 DPM) is considered as the maximum tolerable defect rate of long-term performance. Given this, a shift leading to more than 5% erroneous results is considered a medically important error or a critical error; such as a critical systematic error (SEcrit) calculated as SEcrit = [(TEa – |B|) /SD] – 1.65 (5). Here, the 1.65 represents one-sided z-value for a 5% chance of producing erroneous results. The term (TEa – |B|) / SD is the same Sigma, and therefore the equation is shortened as SEcrit = Sigma_(observed)_ – 1.65 (4). The SQC should be chosen so that it has a Ped ≥ 90% at the SEcrit to give assurance that in long-term the defect rate will not exceed 5%. Given a performance with 5% defect rate is equal to a Sigma of 1.65, the common practice in SQC planning is to assure a long-term quality of 1.65 Sigma. In other words, an Assured Sigma of 1.65 is the target of common SQC procedures; as is reflected in the upper x-axis of the common power curves starting from 1.65 Sigma (Figure 2). It is of importance to note that this does not mean that in long-term the quality will always be 1.65 corresponding to 5% defect rate, but this is the worst case. Meaning that in the presence of SQC, there is a maximum chance of 5% for the results to be incorrect.

For example, given the critical SE for a 6 Sigma method is 4.35 (*i.e.* 6 – 1.65), then the single rule 1:3s N1 with a Ped = 90% at SE = 4.35 can provide an Assured Sigma of 1.65 for a method with Observed Sigma of 6 (Figure 2). In comparison, a method with Observed Sigma of 4 will have a SEcrit of 2.35 (*i.e.* 4 - 1.65), then the multi-rule 1:3s/2:2s/R:4s/4:1s N4 with a Ped = 90% at SE = 2.35 can provide an Assured Sigma of 1.65 for an Observed Sigma of 4 (Figure 2). With lower Observed Sigmas, tougher SQCs are needed to provide the 1.65 Assured Sigma. For example, to control a 3 Sigma method a multi-rule such as 1:3s/2of3:2s/2:2s/R:4s/3:1s/6:x N6 is needed.

## How to provide higher patient safety with high Sigma methods?

**Figure 3:**
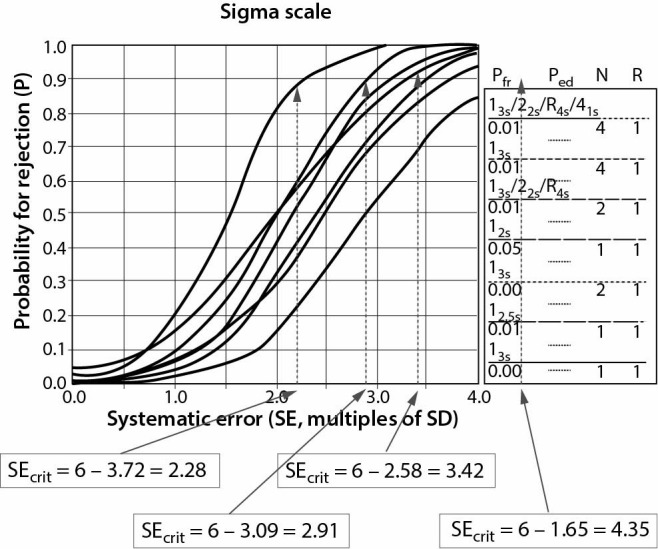
Selecting statistical quality control (SQC) procedures for providing different levels of Assured Sigma for an Observed Sigma of 6. The arrows (left to right) represent critical systematic errors (SEcrit) of 2.28, 2.91, 3.42, and 4.35, which correspond to assured long-term defect rates of 0.01%, 0.5%, and 5%, respectively. Pfr - the probability of false rejection. Ped - the probability of error detection. N - number of quality control material assayed per run. R - number of runs through which the SQC rule is interpreted. (The figure is produced by EZ Rules v.3 software. Westgard QC, Madison, USA).

The improved quality of high Sigma methods can be devoted to two different issues: to facilitate SQC procedures (the laboratory achievement), and/or to assure higher levels of quality for the test results (the patients’ achievement). With a 5% threshold for SQC, the laboratory, in fact, gains more advantage than patients of high Sigma methods. Because high Sigma methods are controlled by cheap and easy single rule SQCs compared to low Sigma methods that must be controlled with tough multi-rule SQCs. To devote the superiority of high Sigma methods to patient safety, the SQC bar should be set higher aiming at assuring defect rates lower than 5%. Figure 3 and Table 2 represent different SQCs needed to provide different levels of Assured Sigma with an Observed Sigma of 6. A long-term defect rate of 5% (50,000 DPM) is easily assured by 1:3s N1. If it is desired to assure a defect rate of 0.5% (5000 DPM), then z (P = 0.995; one-sided) = 2.58 and SQC must be planned to assure a long-term quality of 2.58 Sigma; and therefore, SEcrit = Observed Sigma – 2.58. With a target of 2.58 Sigma as the long-term quality, SEcrit for a 6 Sigma method is 6 – 2.58 = 3.42. Here, the 1:3s N2 has a Ped close to 90% at SE = 3.42, and can be applied to assure the long-term defect rate of 0.5%. It is of note that assuring a long-term defect rate of ≤ 0.5% consumes 2 times more control material than assuring a defect rate of ≤ 5%. If the bar is set even higher to assure less than 0.1% defect rate (1000 DPM); then z (P = 0.999; one-sided) = 3.09, and SEcrit = 6 – 3.09 = 2.91. Providing an Assured Sigma of 3.09 with a 6 Sigma method needs applying 1:3s N4, which consumes 4 times more control material than assuring the long-term Sigma of 1.65. And finally, to assure less than 0.01% defect rate (100 DPM); z (P = 0.9999; one-sided) = 3.72, SEcrit = 6 – 3.72 = 2.28, and SQC is 1:3s/2:2s/R:4s/4:1s N4 which, in addition to higher expense and cumbersome, has a Pfr = 3%. As is seen, more stringent SQCs are needed to provide higher levels of Assured Sigma. Note that even with a very stringent SQC such as 1:3s/2:2s/R:4s/4:1s N4, the long-term defect rate is far higher than the arbitrary assumption in Six Sigma (100 DPM *vs.* 3.4 DPM). Table 3 compares the worst qualities expected from different SQCs with the worst qualities assumed in Six Sigma methodology for different Sigma performances.

**Table 2:** Comparing statistical quality control procedures needed to assure different maximum defect rates with an observed sigma of 6

**Desired long-term DPM**	**Assured Sigma**	**SEcrit**	**SQC procedure**
**50,000**	1.65	4.35	1:3s N1
**5000**	2.58	3.42	1:3s N2
**1000**	3.09	2.91	1:3s N4
**100**	3.72	2.28	1:3s/2:2s/R:4s/4:1s N4
Column 1 is the maximum defect rate that is desired, and column 2 is the corresponding Assured Sigma value. Column 3 presents SEcrit for a 6 Sigma method calculated as 6 minuses Assured Sigma (*e.g.* for the last row: 6 - 3.72 = 2.28). DPM - defects *per* million. SEcrit - critical systematic error. SQC - statistical quality control. N - number of control materials assayed *per* run.			

**Table 3:** The expected long-term defect rate for different short-term Sigmas

Observed/Short-term Sigma (Defect rate, DPM)*	**Assured/Long-term Sigma (long term defect rate, DPM)**							
**Six Sigma**	**SQC1**	**SQC2**	**SQC3**	**SQC4**	**SQC5**	**SQC6**	**SQC7**
**6 (0.002)**	4.5 (3.4)	3.8 (100)	3.1 (1000)	2.9 (1900)	2.7 (3500)	2.5 (6200)	2.3 (11,000)	1.7 (44,000)
**5 (0.6)**	3.5 (230)	2.8 (3000)	2.1 (18,000)	1.9 (29,000)	1.7 (45,000)	1.5 (67,000)	1.3 (97,000)	0.7 (242,000)
**4 (63)**	2.5 (6200)	1.9 (45,000)	1.1 (136,000)	0.9 (184,000)	0.7 (242,000)	0.5 (308,000)	0.3 (382,000)	- 0.3 (618,000)
**3 (2700)**	1.5 (66,800)	0.7 (242,000)	0.1 (460,000)	- 0.1 (540,000)	- 0.3 (620,000)	- 0.5 (690,000)	- 0.7 (760,000)	- 1.3 (900,000)
**2 (45,500)**	0.5 (308,000)	- 0.3 (620,000)	- 0.9 (816,000)	- 1.1 (864,000)	- 1.3 (900,000)	- 1.5 (933,000)	- 1.7 (955,000)	- 2.3 (990,000)
DPM - defect per million. Column 1 presents Observed Sigma calculated from short-term evaluation data, and the defect rate. Column 2 presents the long-term defect rate assuming the arbitrary 1.5 Sigma shift assumed by Six Sigma. Columns 3-9 present long-term defect rate expected with a probability of 90% when different SQCs are applied to control a certain Observed Sigma. SQC1 to SQC7 correspond to SQC procedures presented in Figure 2; SQC1: 1:3s/2:2s/R:4s/4:1s N4; SQC2: 1:3s N4; SQC3: 1:3s/2:2s/R:4s N2; SQC4: 1:2s N1; SQC5: 1:3s N2; SQC6: 1:2.5s N1; SQC7: 1:3s N1. Note that defect rates in column 3 are higher than corresponding defect rate in column 2, meaning that even SQC1 (the toughest SQC presented in Figure 2) cannot assure the long-term defect rate assumed by Six Sigma because a shift of 2.3 SD is needed for SQC1 to reach Ped = 90%, while the maximum shift assumed in Six Sigma is 1.5 SD.								

## Discussion

Laboratory results, to be helpful in making correct medical decisions, must be of good reliability. The least reliability that can be attributed to the results is determined by the maximum defect rate expected for long-term performance. Practically it is not possible to determine the defect rate via long-term evaluations, so methods are evaluated in short periods and then an assumption is made about the maximum defect rate that is expected in long-term.

Based on Six Sigma, the worst case scenario is that long-term Sigma is 1.5 Sigma less than short-term Sigma. J. O. Westgard, who first introduced the Sigma equation for the medical laboratory application, applies the Six Sigma approach for calculating the long-term defect rate (*4*). In the Westgard approach, Sigma is calculated from the short-term data of bias and imprecision, then to convert the calculated Sigma to long-term defect rate, Sigma tables are consulted in which defect rates are not presented exactly for the calculated Sigma but for the “calculated Sigma – 1.5”.

The Westgard approach to estimate the long-term defect rate is in compliance with the Six Sigma methodology, and does not consider the capability of the control process. In contrast to the Six Sigma assumption, analytical performance in the medical laboratory is prone to instabilities larger than 1.5 Sigma that, in addition, do not spontaneously return to the stable state. Therefore, control procedures are applied to detect shifts before affecting patient safety. Commonly, SQC procedures are planned to detect shifts that are large enough to produce more than 5% erroneous results (*5*). With a SQC goal of < 5% defect rate, irrespective of the observed quality in short-term, the quality that is assured for long-term is 1.65 Sigma. By sure, a high Sigma method when stable or in the case of shifts smaller than the size rejected by SQC, performs with a quality better than 1.65 Sigma. But there is no mean to determine the shift size at any certain moment; therefore, as long as the SQC has not rejected the performance we can only give the assurance that the defect rate has not yet exceeded 5% corresponding to 1.65 Sigma. It is of importance to note that the improved quality of high Sigma methods can be devoted to facilitate SQC procedures and/or to assure higher levels of quality for the test results. As long as the target of SQC is an Assured Sigma of 1.65, applying high Sigma methods does not add so much to patient safety because the improved Sigma is mostly devoted to easing the SQC procedure. If it is intended to improve patient safety, SQC should aim at higher levels of Assured Sigma; meaning applying more stringent SQCs. Woodworth *et al.* present an example for the relationship between the long-term defect rate and the capability of the quality control procedure (*6*). Evaluating long-term defect rates expected for several HbA1c methods, they show that applying stringer SQCs leads to decreased number of erroneous results, and conclude that: “(Observed) Sigma values are directly related to the predicted probability of producing unreliable patient results during stable operation, while the maximum expected erroneous results during an out of control operation is determined by the SQC strategy used by the laboratory”. Thus, they recommend that evaluation of the SQC plan must be included in the process of estimating the expected risk of the analytical phase in long-term performance (*6*). The last edition of the Clinical and Laboratory Standards Institute (CLSI) C24 guideline addresses how a laboratory’s QC practices affect the risk of patient harm in long-term performance (*7*).

Recently the International Federation of Clinical Chemistry and Laboratory Medicine (IFCC) Task Force on Implementation of HbA1c standardization (TF-HbA1c) has set default risk levels of 2 Sigma for routine laboratories and, to provide more reliable data to be used in providing clinical practice guidelines, 4 Sigma for laboratories performing clinical trials (*8*). This means that different levels of Assured Sigma for routine and clinical trial applications should be instituted. Therefore, the same method when used in clinical trials should be controlled with a more stringent SQC than when used in routine laboratories. For example, with a 6 Sigma method, 1:2.5s N1 SQC is enough to provide the Assured Sigma of 2 in the routine application while the multi-rule 1:3s/2:2s/R:4s/4:1s N4 must be applied to provide the Assured Sigma of 4 in the clinical trial application.

By sure, the ultimate goal of the quality assurance in the medical laboratory is to achieve the highest possible level of patients’ safety. If it is desired to provide high levels of Assured Sigma by simple SQC procedures, the Observed Sigma should be substantially greater than the desired Assured Sigma to provide enough space needed for simple SQCs to reach a Ped ≥ 90%. For example, 1:3s N1, the simplest SQC in the collection presented in Figure 2, needs a 4.35 Sigma shift before it reaches Ped = 90%. Therefore, to apply this SQC, the Observed Sigma must be at least 4.35 Sigma higher than the desired Assured Sigma. For example, to assure a long-term quality of < 3.4 DPM (Assured Sigma = 4.5), the Observed Sigma should be ≥ 8.85 (*i.e.* 4.5 + 4.35). Recently Erna Lenters-Westra and Emma English have evaluated 3 new HbA1c methods - here called methods A, B, and C - and have determined their Sigmas as 8.6, 6.9, and 3.3, respectively (*9*). Given the TF-HbA1c goals of 2 and 4 Sigma respectively for routine application and clinical trials, method A can be controlled easily by 1:3s N1 in both routine laboratories and clinical trials to provide both Assured Sigmas of 2 and 4, respectively. Method B can still be controlled by 1:3s N1 in the routine application, but in clinical trials, it should be controlled by a more stringent SQC e.g. 1:3s N4. Method C with Sigma of 3.3 is not acceptable for clinical trials because its quality is less than the target of 4 Sigma. On the other hand, controlling this method to provide an Assured Sigma of 2 in routine laboratory needs applying a complex multi-rule SQC such as 1:3s/2of3:2s/R:4s/3:1s/6x N6, which, in addition to high expense and trouble, has a Pfr of 5%.

The 1:3s/2of3:2s/R:4s/3:1s/6x N6 SQC procedure, one of the toughest practical SQCs, needs a systematic shift of 1.7 Sigma to reach Ped = 90%. So, to assure less than 5% erroneous results in long-term, the least needed Observed Sigma is 3.35 Sigma (1.65 + 1.7). Therefore, although methods with Observed Sigmas of 1.65 to 3.35 are theoretically acceptable, such methods are not practically controllable. This is the basis for the advice frequently emphasized by J. O. Westgard that methods with qualities of < 3 Sigma should not be used in medical laboratories because these methods cannot be controlled by practically affordable SQC procedures (*5*). Similarly, with higher goals of Assured Sigma, the least Observed Sigma that can be controlled by these SQCs must be again 1.7 Sigma higher than the goal. For example, with the 4 Sigma goal set by TF-HbA1c for clinical trials, a HbA1c method must have an Observed Sigma of ≥ 5.7 (4 + 1.7) to be controllable in clinical trials. Thus, although a HbA1c method with Observed Sigma of *e.g.* 4.5 is theoretically acceptable for clinical trials, there is no practical SQC to assure a long-term Sigma of 4 suggested by TF-HbA1c. To repeat J. O. Westgard advice, HbA1c methods with qualities of < 5.7 Sigma should not be used in clinical trials.

Interestingly, the 1.7 Sigma shift needed for 1:3s/2of3:2s/R:4s/3:1s/6x N6 is close to the 1.5 Sigma shift assumed in the Six Sigma. The important difference is that the Six Sigma assumes that the 1.5 shift is the worst scenario even with no QC applied, while the 1.7 shift in medical laboratory is the best scenario with applying the most stringent SQC. If looser SQCs are applied, shifts larger than 1.7 Sigma is expected; and with no QC, shifts of any size are expected.

Since Sigma values are related to the area under the curve of the Gaussian distribution, it maybe seem that Sigma values cannot be treated as linear parameters, *e.g.* in the equation Sigma_(Assured)_ = Sigma_(Observed)_ - Sigma_(SQC)_. Given the Sigma value is nothing but the number of the SDs that fit in the distance between mean and the nearest TEa limit, any value that is in the SD units can be added to or subtracted from Sigma as linear parameters. For example, if a bias of 2 SD happens for a bias-free 5-Sigma method, then the method becomes a 3-Sigma method because the shift has subtracted 2 SDs from the original Sigma; simply calculated as 5 – 2 = 3. It is the same for changes in the imprecision; for example, if the imprecision of a 6-Sigma method triples, then the method becomes a 2-Sigma method (calculated as 6 ÷ 3 = 2); and if the imprecision of this method halves, the new Sigma becomes 12 (calculated as 6 × 2 = 12). The thing that cannot be treated as linear parameter is the defect rate, because there isn’t a linear relation between Sigma value and defect rate. For example, if Sigma is halved, it cannot be concluded that the defect rate is also halved, but the new defect rate must be calculated as the area under the Gaussian curve corresponding the new Sigma.

Coskun *et al.* have criticized the Westgard approach to Sigma calculation (*10*). They claim that the Westgard approach is one-sided and 50% of data are not included in the performance calculation, therefore the Sigma level is lower than the actual Sigma; and they recommend that the actual Sigma level can be calculated using the area under the curve, what they call ‘a new approach to calculating Sigma metric’ (*10*). The fact is that Sigma values and defect rates are anywhere converted to each other using the area under the Gaussian curve, and the approach recommended by Coskun *et al.* is not something new. In the Westgard approach, the Sigma scores are converted to the defect rates using Sigma tables that are produced based on the area under the curve, and, therefore, by no means 50% of the data are missed from the performance calculation. The problem with the Coskun *et al.* is that they have confused the short-term/long-term concept with the one-sided/two-sided concept. The reason that the calculated defect rates in the Coskun *et al.* calculations are significantly lower than the Westgard approach is that they have neglected the 1.5 SD subtraction. Coskun *et al.* in fact, have calculated the short-term/Observed defect rates, whereas in the Westgard approach (that is in compliance with the Six Sigma approach) 1.5 SD is subtracted from the calculated Sigma and therefore the long-term/Assured defect rates are calculated which are higher than the short-term/Observed defect rates.

To conclude, long-term Sigma is a measure of the reliability of laboratory results. In contrast to the Six Sigma methodology, the long-term quality in the medical laboratory is not arbitrarily 1.5 Sigma less than the short-term quality, but is determined by the shift needed for the applied SQC to reject the performance with a high Ped. In other words, the long-term quality of analytical performance in the medical laboratory is the quality that can be assured with the applied SQC. This vein, long-term Sigma can be called Assured Sigma. Given the Six Sigma tables present long-term defect rates assuming 1.5 Sigma shift, referring to these tables to determine the long-term defect rate of analytical performance in the medical laboratory will be misleading. To calculate long-term defect rate of analytical performance, laboratorians must calculate the shift needed for the SQC to reach a Ped of 90%, Sigma_(SQC)_; subtract Sigma_(SQC)_ from Observed Sigma to calculate Assured Sigma; and finally calculate long-term defect rate as P (z > Assured Sigma).
